# Quantifying welfare impairment from respiratory disease in fattening pigs using adaptive conjoint analysis

**DOI:** 10.1186/s40813-026-00533-1

**Published:** 2026-08-14

**Authors:** Marloes Boeters, Beatriz Garcia-Morante, Tijs Tobias, T. Bas Rodenburg, Carla Correia-Gomes, Joaquim Segalés, Maria Costa, Jonathan Rushton, Gerdien van Schaik, Wilma Steeneveld

**Affiliations:** 1 Department of Population Health Sciences, division Farm Animal Health, Faculty of Veterinary Medicine, Utrecht University, Utrecht, The Netherlands; 2 IRTA, Animal Health, Centre de Recerca en Sanitat Animal (CReSA), Campus Universitat Autònoma de Barcelona (UAB), Bellaterra, Catalonia 08193 Spain; 3 Unitat Mixta D’investigació IRTA-UAB en Sanitat Animal, Centre de Recerca en Sanitat Animal (CReSA), Campus Universitat Autònoma de Barcelona (UAB), Bellaterra, Catalonia 08193 Spain; 4 WOAH Collaborating Centre for the Research and Control of Emerging and Re-Emerging Swine Diseases in Europe (IRTA-CReSA), Bellaterra, Catalonia 08193 Spain; 5 Royal GD, Deventer, The Netherlands; 6 Department of Population Health Sciences, Animals in Science and Society, Faculty of Veterinary Medicine, Utrecht University, Utrecht, The Netherlands; 7 Animal Health Ireland, Carrick-on-Shannon, Ireland; 8 Departament de Sanitat i Anatomia Animals, Facultat de Veterinària, Campus Universitat Autònoma de Barcelona (UAB), Bellaterra, Catalonia 08193 Spain; 9 Centre for Epidemiology and Planetary Health, School of Veterinary Medicine and Biosciences, SRUC (Scotland’s Rural College), Inverness, UK; 10 GBADs, Roslin Institute, University of Edinburgh, Easter Bush Campus, Midlothian, EH25 9RG UK

**Keywords:** Animal welfare, Five Domains Model, Swine, Survey, PRDC, Expert elicitation

## Abstract

Respiratory disease remains highly prevalent in pig production and constitutes a major health and welfare challenge worldwide. However, most studies assessing pig welfare impairment from respiratory disease rely on isolated animal-based indicators, limiting integration across welfare domains. This study aimed to quantify welfare impairment from respiratory disease in fattening pigs (8–10 weeks of age) using indicators aligned with the Five Domains Model. A survey using adaptive conjoint analysis was conducted with animal welfare researchers (*n* = 32) and practising swine veterinarians (*n* = 22). Respondents completed pairwise comparisons of profiles defined by combinations of five welfare indicators—appetite, coughing, respiratory distress, play behaviour, and exploratory behaviour—each expressed at increasing severity levels. Based on respondents’ choices of which profile represented greater welfare impairment, indicator importance scores and level-specific contributions to pig welfare impairment from respiratory disease were estimated. Across respondent groups, severe respiratory distress was perceived as the largest contributor to pig welfare impairment from respiratory disease, with an estimated contribution of 29.8%, followed by repeated coughing (+ 22.7%) and complete loss of appetite (+ 19.2%). Reductions in play and exploratory behaviour were perceived to contribute comparatively less to welfare impairment from respiratory disease but remained relevant. Agreement between researchers and veterinarians was high, with statistically significant differences limited to occasional coughing and complete loss of play behaviour. In addition, profiles representing single-pathogen respiratory infections were constructed based on expert elicitation, providing an illustrative demonstration of how outcomes from adaptive conjoint analysis may be used to assess welfare impairment under different respiratory disease scenarios. The findings of this study may inform refinement of welfare assessment protocols, support the integration of welfare metrics into epidemiological models, and enable comparison of disease scenarios. However, results should be interpreted with caution, as they reflect perceived rather than directly measured welfare impairment and assume independence between biologically interrelated indicators. Future work should prioritise the application of welfare impairment estimates to biologically realistic welfare profiles in which interrelated indicators co-occur, while also exploring integration with observational approaches and extension to broader, multifactorial welfare contexts.

**Supplementary Information:**

The online version contains supplementary material available at https://doi.org/10.1186/s40813-026-00533-1.

## Background

Respiratory disease remains highly prevalent in pigs and constitutes a major health and welfare challenge worldwide, primarily by affecting fattening pigs. The most common viral contributors to respiratory disease include porcine reproductive and respiratory syndrome virus (PRRSV), swine influenza virus (SIV), and porcine circovirus 2 (PCV2), while major primary bacterial pathogens are *Mycoplasma (M.) hyopneumoniae* and *Actinobacillus (A.) pleuropneumoniae* [1]. Co-circulation of these pathogens can result in complex and potentially synergistic interactions, with management and environmental factors further modulating disease expression and severity. This leads to heterogeneous disease manifestations and a large variation in the magnitude and nature of the associated welfare impact [2].

Pig welfare can be systematically conceptualised using the Five Domains Model, which builds on and extends the Five Freedoms framework, distinguishing four physical domains—health, nutrition, environment, and behaviour—and their combined influence on the animal’s mental state [3]. Of the physical domains, respiratory disease primarily compromises the health, nutrition, and behaviour domains, while the environmental domain plays a key role in shaping disease risk and severity rather than being directly affected by respiratory disease itself. Within the health domain, respiratory disease is commonly associated with lesions of the respiratory system such as (broncho-)pneumonia, pleurisy or tracheitis, and clinical signs such as hyperthermia, coughing, and respiratory distress [1, 4]. Impairment of the nutrition domain may occur through reduced appetite and feed intake [5]. Although less frequently investigated, respiratory disease may also affect the behaviour domain by disrupting normal behavioural patterns, including play and exploratory behaviour [6]. Collectively, impairments across the health, nutrition, and behaviour domains may translate into adverse mental experiences such as malaise, breathlessness, weakness, exhaustion, or negative affective states (e.g. boredom, discomfort, stress, fatigue, fear, frustration and pain), to varying degrees [3].

Although the impact of respiratory disease on pig welfare has been acknowledged [1, 7], most studies assess welfare using isolated indicators from a single domain, such as coughing frequency or lung lesion prevalence (at slaughter) within the health domain [8, 9]. The single-domain approach limits comparability across studies assessing the welfare impact of different manifestations of respiratory disease in individual pigs as well as comparisons across farms and regions. Consequently, the prioritisation of control strategies when multiple welfare dimensions are affected simultaneously becomes complicated. Therefore, welfare impairment should ideally be assessed across all relevant welfare domains and expressed using a single, integrative metric. A key challenge in developing such a metric, however, lies in determining the relative contribution of impairments in different domains to overall welfare, as these contributions are unlikely to be equal. Assigning appropriate weights to welfare indicators is therefore essential, yet cannot be derived from empirical measurements alone.

Expert elicitation methods are widely used in animal welfare research to address this challenge by capturing informed judgements regarding the relative importance of welfare impacts, often with the aim of identifying, weighting, and prioritising key welfare issues [10, 11]. Recently, survey-based adaptive conjoint analysis (ACA) has been demonstrated as a promising method for estimating disease-related welfare impairment by systematically integrating expert preferences [12, 13]. In contrast to traditional Delphi-based approaches [e.g. 11], which rely on iterative consensus-building through group discussion, ACA allows experts to directly evaluate trade-offs between different welfare impacts. This provides a structured and transparent estimate of the relative contribution of individual welfare indicators to overall welfare impairment, while reducing the influence of group dynamics.

The aim of the current study was to quantify welfare impairment from respiratory disease in fattening pigs (8–10 weeks of age) through the evaluation of five key welfare indicators aligned with the Five Domains Model, using an ACA approach. In addition, we aimed to consider differences in perceptions between animal welfare researchers and practising swine veterinarians, as perceptions of welfare impairment may vary depending on practical experience and scientific focus. As an illustration of how ACA-derived outcomes could be applied, we subsequently used the outcomes to assess the perceived welfare impairment from respiratory disease associated with five single respiratory pathogen infections: PRRSV, SIV, PCV2, *M. hyopneumoniae* and *A. pleuropneumoniae*.

## Methods

### Adaptive conjoint analysis for quantifying welfare impairment

Originally developed and widely used in marketing and health economics, ACA is grounded in the assumption that individuals assign a part-worth utility to each level of an attribute (i.e. a characteristic of a product, such as brand or colour), reflecting its relative importance. The total utility of a product comprising several attributes is then calculated as the sum of part-worth utility values associated with its specific attribute levels. In a typical application, higher summed utilities reflect stronger preference for a particular option [14]. In our study, the attributes were welfare indicators derived from the three relevant physical domains: nutrition, health, and behaviour. Because the study context concerned welfare impairment rather than consumer preference, part-worth utilities were elicited and interpreted as disutilities, with their sum representing the perceived undesired welfare impairment associated with a specific combination of welfare indicator levels. Accordingly, higher summed part-worth disutilities reflected greater perceived welfare impairment. For a more detailed description of the development and implementation of the ACA method, readers are referred to Edwardes et al. [13].

### Selection of welfare indicators and their levels

Pig health and welfare researchers and practising veterinarians were consulted in separate meetings to identify welfare indicators that were as independent as possible, together with mutually exclusive indicator levels representing the range of severity observed in fattening pigs affected by respiratory disease. Because complete independence among welfare indicators is not achievable in practice, four pig health experts (authors BGM, TT, CCG, and JS) subsequently evaluated the proposed indicators to identify the most independent set attainable. The final indicators were selected because they can plausibly vary independently in occurrence or severity, although they remain biologically interrelated manifestations of respiratory disease. Consequently, as ACA assumes independence between attributes, the estimated part-worth disutilities should not be interpreted as independent causal effects on welfare impairment, but rather as perceived marginal contributions within the conjoint framework used in this study.

The final set of welfare indicators and their corresponding levels is presented in Table 1. The number of welfare indicators and the number of levels per indicator were limited to a maximum of five and four, respectively, to ensure feasibility for respondents and reliable estimation of part-worth disutilities [15].

**Table 1:** Selected welfare indicators associated with respiratory disease across animal welfare domains^1^, including their levels and descriptions

Domain	Welfare indicator	Levels	Description	Reference
Nutrition	Appetite	1. Normal (optimal) 2. Moderate decrease 3. Total loss	Normal appetite - feed use is apparent Pig shows partial feed consumption No visible evidence of feed intake	Steinerová et al. [6], Trindade et al. [27]
Health	Coughing	1. Absence (optimal) 2. Occasional 3. Short bouts 4. Repeated coughing	No coughing Sporadic coughs, easily countable Short bouts, followed by periods without coughing Continual or repeated marked coughing (not bouts)	Steinerová et al. [6], Roberts et al. [28]
	Respiratory distress	1. Absence (optimal) 2. Mild 3. Moderate 4. Severe	Normal respiratory rate of 25–40 breaths per min without abdominal effort. Increased respiratory rate (> 40 breaths/min) without abdominal effort. Increased respiratory rate (> 40 breaths/min) with slight abdominal effort. Increased respiratory rate (> 40 breaths/min) with obvious abdominal breathing, open-mouth breathing, grunting, and/or dog-sitting posture.	Steinerová et al. [6], Roberts et al. [28]
Behaviour	Play behaviour (locomotor and social play)^2^	1. Normal (optimal) 2. Moderate decrease 3. Total loss	Normal (assume the pig spends 2 min of the 10 min playing during observation after feeding) Halved No visible evidence of play behaviour	Steinerová et al. [6], Steinerová et al. [29]
	Exploratory behaviour^3^	1. Normal (optimal) 2. Moderate decrease 3. Total loss	Normal (assume the pig spends 6 min of the 10 min exploring during observation after feeding) Halved No visible evidence of exploratory behaviour	Steinerová et al. [6], Steinerová et al. [29]

Indicators and their levels were selected through expert consultation to identify the most independent feasible set of welfare indicators and mutually exclusive levels representing the range of respiratory disease severity in fattening pigs (8–10 weeks of age). ^*1*^*Mellor (2017);* ^*2*^*Energetic movements like running or jumping*,* alone or with others; includes mutual pushing and head-knocking without aggression.* ^*3*^*Interaction with inanimate objects by rooting*,* nudging*,* sniffing*,* licking*,* chewing*,* or pushing*

### Survey design

**Fig. 1:**
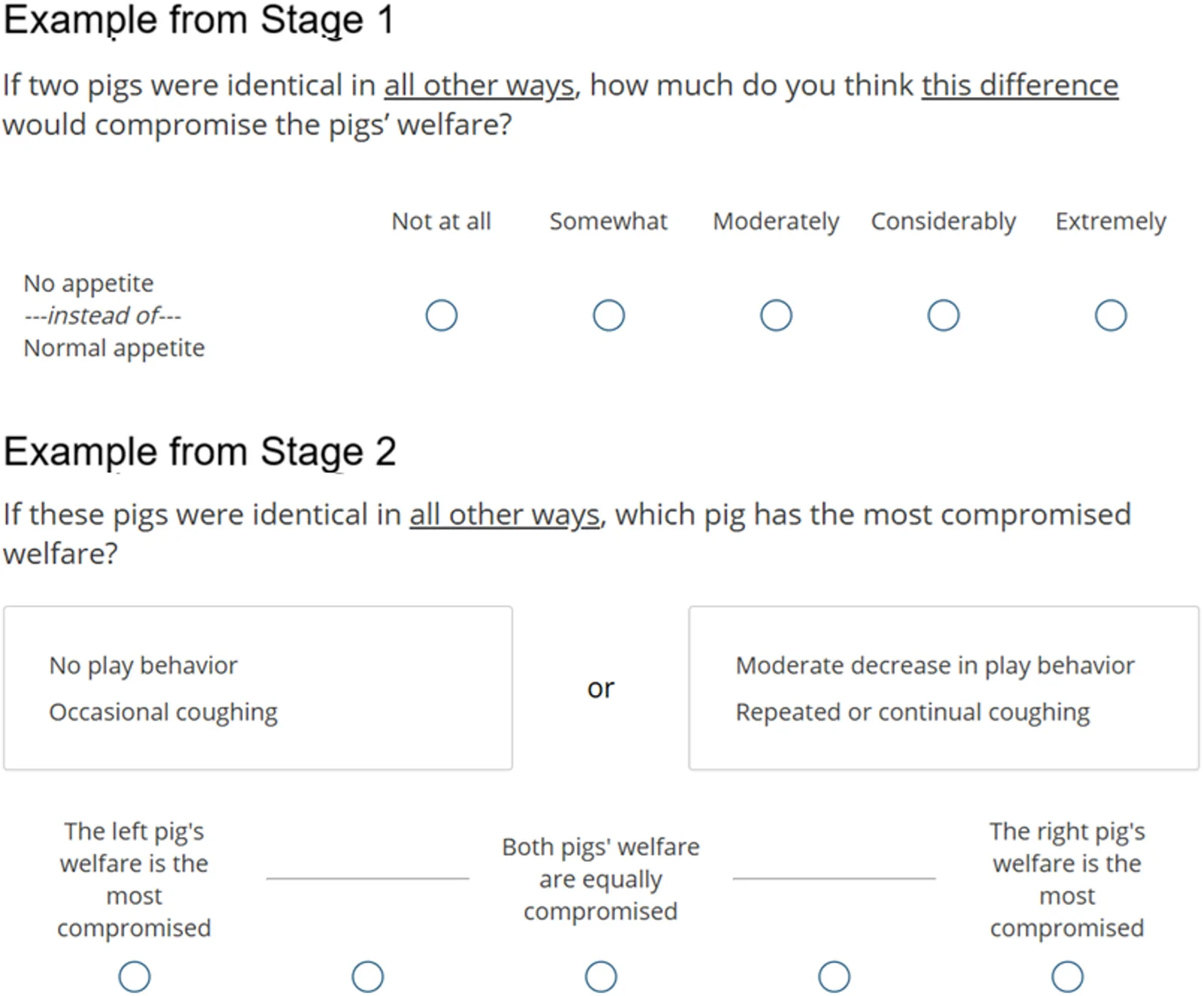
Example questions for each stage of the adaptive conjoint analysis survey. Stage 1: indicator-level importance questions. Stage 2: paired-comparison questions

The ACA survey was developed using Lighthouse Studio [16]. Figure 1 provides example questions for each stage of the survey as will be described below. To guide the ACA algorithm, we specified a priori assumptions such that higher levels of each welfare indicator would correspond to greater welfare impairment. For example, repeated coughing (level 4) was assumed to impair welfare more than short bouts of coughing (level 3), which in turn was expected to impair welfare more than occasional or no coughing (levels 2 and 1, respectively).

Before completing the ACA tasks, respondents answered background questions regarding country of residence, age, gender, highest level of education, primary professional role, and experience working with pigs and animal welfare. Respondents were then instructed to imagine standing in front of a pen on a conventional pig farm and observing two fattening pigs (8–10 weeks of age) during feeding and for 10 min thereafter. Both pigs were affected by respiratory disease to varying degrees, and respondents were told to assume that any deviation from the optimal level (normal functioning) of a welfare indicator was entirely attributable to respiratory disease rather than to random variation or other causes. Respondents were provided with the definitions of all welfare indicators and levels presented in Table 1, and then completed the ACA procedure, which consisted of two stages (Fig. 1).

#### Stage 1. Assessing indicator-level importance

Respondents first assessed the overall importance of each welfare indicator by rating how much the change from its lowest (optimal) to highest (most severe) level would compromise pig welfare, using a 5-point scale ranging from “not at all” to “extremely.” These initial ratings provided respondent-specific preliminary estimates of the part-worth disutilities for each indicator and guided the ACA algorithm to focus on the differences that the respondent considered most meaningful, thereby improving the efficiency and precision of subsequent trade-off evaluations.

#### Stage 2. Paired-comparison questions

In the second stage, respondents completed twelve paired-comparison trade-off questions. Prior to survey implementation, three pig health experts (authors BGM, TT, CCG) reviewed possible combinations of welfare indicator levels and identified those that were highly unlikely to occur in practice. These unlikely combinations were excluded to avoid presenting respondents with unrealistic welfare scenarios. An overview of excluded combinations is provided in Supplementary File Table 1.

Every paired-comparison question presented two “virtual pigs,” each characterised by a specific combination of two or three welfare -indicator levels. The respondent then had to choose which pig’s welfare was most impaired. For each question, the ACA algorithm generated a pair that was predicted to have similar overall welfare impairment based on their interim sums of part-worth disutilities. After each response, part-worth disutilities were updated using ordinary least squares regression, and the updated estimates informed subsequent questions. This adaptive sequence allowed the survey to focus on the most informative trade-offs for each respondent.

### Recruitment of respondents

We recruited two respondent groups: animal welfare researchers and practising swine veterinarians. Animal welfare researchers were invited through flyers distributed at the 58th Congress of the International Society for Applied Ethology, held in Utrecht, The Netherlands. As this congress was also attended by policy makers, students, and other delegates, eligibility was restricted to individuals working as animal welfare researchers; respondents not meeting this criterion were excluded. Veterinarians were recruited by email through the professional networks of the authors. Eligibility for this group required respondents to be practising veterinarians with experience working with pigs. Two respondents were also involved in the expert consultation during indicator selection (authors TBR and TT); however, they completed the survey in their professional capacity as an animal welfare researcher and practising veterinarian, respectively. Finally, respondents from either group who completed the survey in less than five minutes were excluded, as pilot testing indicated this was the minimum time required to complete the questionnaire.

### Data processing and analysis

#### Analysis of ACA-derived disutilities and relative importance scores

The ACA analysis software (ACA 5.2.2; [16]) generated estimates of each participant’s part-worth disutilities for every welfare indicator level using hierarchical Bayes (for technical details, see: Sawtooth Software [17]). Final disutilities were exported and statistical analyses were conducted in R (version 4.5.0; [18]).

We derived the relative importance score of each welfare indicator for each respondent, by taking the range of part-worth disutilities for each indicator, dividing it by the sum of ranges across all welfare indicators for that respondent, and expressing the result as a percentage [14, 17]. We then calculated the mean and 2.5–97.5th percentile range of these relative importance scores across respondents. Differences in perceived welfare impairment between researchers and veterinarians were explored using Mann–Whitney U tests for each indicator level, with statistical significance set at 0.05. To examine differences across levels of pig-related experience and perceived importance of animal welfare, Kruskal–Wallis tests were conducted, with p-values adjusted for multiple testing using the Benjamini–Hochberg procedure.

#### Development and application of a 0-100% welfare impairment from respiratory disease scale

To enhance interpretability, we chose to express welfare impairment from respiratory disease on a 0–100% scale, rather than presenting the raw disutility values derived from ACA, as these disutility values lack a clear and meaningful unit of interpretation. It is important to emphasise that this 0–100% scale is conditional on the respiratory disease indicators included in this study. However, for simplicity, we refer to this construct as “welfare impairment from respiratory disease”, while emphasising that it is a relative, indicator-based measure and not a general or absolute measure of pig welfare or respiratory disease burden.

The scale was constructed by first calculating the total disutility for two reference profiles: (i) the optimal profile, obtained by summing the part-worth disutilities corresponding to the optimal (first) level of each indicator, and (ii) the most severe profile, obtained by summing the part-worth disutilities corresponding to the most severe (last) level of each indicator. These two profiles define the anchors of the scale and correspond to 0% and 100% welfare impairment from respiratory disease (within the indicator set), respectively.

Subsequently, two intermediate scenarios representing mild and moderate respiratory disease were constructed. The moderate scenario was defined by selecting the second highest (second most severe) level for each indicator, whereas the mild scenario was defined by selecting the next lower level. An overview of the selected levels for each severity scenario is provided in Supplementary File Table 2. In addition, the impact of changes in individual welfare indicators was assessed by deteriorating one indicator at a time while holding all others at their optimal levels.

For each evaluated profile—whether representing a predefined severity scenario (mild or moderate) or a single-indicator deterioration—the resulting increase in total welfare impairment from respiratory disease was obtained by linear rescaling between the two reference points:

$$\begin{aligned}&\:Welfare\:impairment\:profil{e}_{i}\cr&\quad =\frac{{U\:optimal}_{i}-U\:profil{e}_{i}}{{U\:optimal}_{i}-{U\:most\:severe}_{i}}\times\:100\%\:\end{aligned}$$

where:

- $\:{U\:optimal}_{i}$ is the total disutility of the optimal welfare profile for respondent *i*, obtained by summing the part-worth disutilities of all optimal (first) levels;
- $\:{U\:most\:severe}_{i}$ is the total disutility of the most severe welfare profile for respondent *i*, obtained by summing the part-worth disutilities of the most severe (last) levels across all indicators;
- $\:{U\:profile}_{i\:}$ is the total disutility of an evaluated welfare profile for respondent *i*. The evaluated profile may represent (i) a scenario in which one indicator is deteriorated while all others remain at their optimal level, or (ii) a predefined mild or moderate respiratory disease scenario combining multiple deteriorated levels.

### Illustrative exercise: perceived welfare impairment by single pathogen infections

To illustrate the potential application of ACA-derived disutilities, five pig health experts (authors BGM, TT, CCG, MC, JS) contributed to the construction of welfare profiles representing the most likely expression and plausible range of disease manifestations associated with single infection with PRRSV, SIV, PCV2, *M. hyopneumoniae* or *A. pleuropneumoniae* in an individual pig at a single moment in time.

To ensure a consistent interpretation across experts, the following assumptions were agreed upon prior to assessment: only circulating, endemic strains in Europe were considered; for PRRSV, this was further specified as a non-highly pathogenic variant of PRRSV-1; and for PCV2, the typical situation was defined as in a population of vaccinated pigs, as most pig farms in Europe routinely vaccinate piglets against this disease. Experts completed this task independently and without access to any results derived from the ACA. The resulting overview of level selection is provided in Supplementary File Table 3.

For each pathogen-specific scenario, the median welfare indicator levels across experts were identified and linked to the corresponding part-worth disutilities (averaged across all respondents). Disutilities were then summed to derive an overall welfare impairment score for each single-pathogen infection. These scores were subsequently rescaled to the same 0–100% range as described in the previous section, to estimate the most likely, minimum, and maximum welfare impairment associated with each pathogen infection.

## Results

### Descriptive information on respondents

Of the 60 complete responses received, six were excluded: five due to ineligible professional role and one due to completing the survey in less than five minutes (veterinarian). This resulted in a final sample of 54 respondents (32 animal welfare researchers and 22 practising swine veterinarians). Table 2 presents descriptive characteristics of the respondents, stratified by professional role.

Among the 32 researchers, the majority were female (*n* = 27), worked with pigs either sometimes or regularly (*n* = 19), and reported animal welfare as the central focus of their research (*n* = 21). Most of the researchers were based in Europe (*n* = 25), with additional respondents from the United States (*n* = 3), Australia (*n* = 2), and Brazil (*n* = 2). Among the 22 veterinarians, most were male (*n* = 13), all reported working regularly with pigs, and most indicated that they continuously consider animal welfare in their professional activities (*n* = 15). Most veterinarian respondents were based in Europe as well (*n* = 21), with one respondent based in the United States.

**Table 2:** Descriptive information on survey respondents, grouped by professional role

Descriptives	Animal welfare researchers	Practising swine veterinarians
Total respondents	32	22
Home country		
Australia	2	0
Austria	1	0
Belgium	1	0
Brazil	2	0
Denmark	2	0
France	2	0
Germany	6	0
Ireland	0	2
North Macedonia	1	0
Netherlands	6	8
Norway	1	0
Spain	1	6
Sweden	1	0
UK	3	5
USA	3	1
Age (years)		
Average	39	48
SD	12.2	13.7
Min-max	23–70	24–78
Gender		
Male	5	13
Female	27	9
Frequency of working with pigs		
Regularly	16	22
Sometimes	3	0
Never	13	0
Experience in animal welfare research/Consideration of animal welfare		
None/Not at all	0	0
Limited/A little	1	0
Moderate/Sometimes	4	1
Substantial/A lot	6	6
Central focus of research/All the time	21	15

### Perceived welfare impairment by indicator

Welfare indicators within the health domain were assigned the highest relative importance scores, indicating that the most severe levels within these indicators resulted in the largest contribution to welfare impairment from respiratory disease, relative to the other indicators (Table 3). Overall, the respiratory distress indicator was assigned the highest importance, followed by coughing, appetite, exploratory behaviour, and play behaviour. Both researchers and veterinarians showed strong agreement in their ranking of the importance of welfare indicators.

**Fig. 2:**
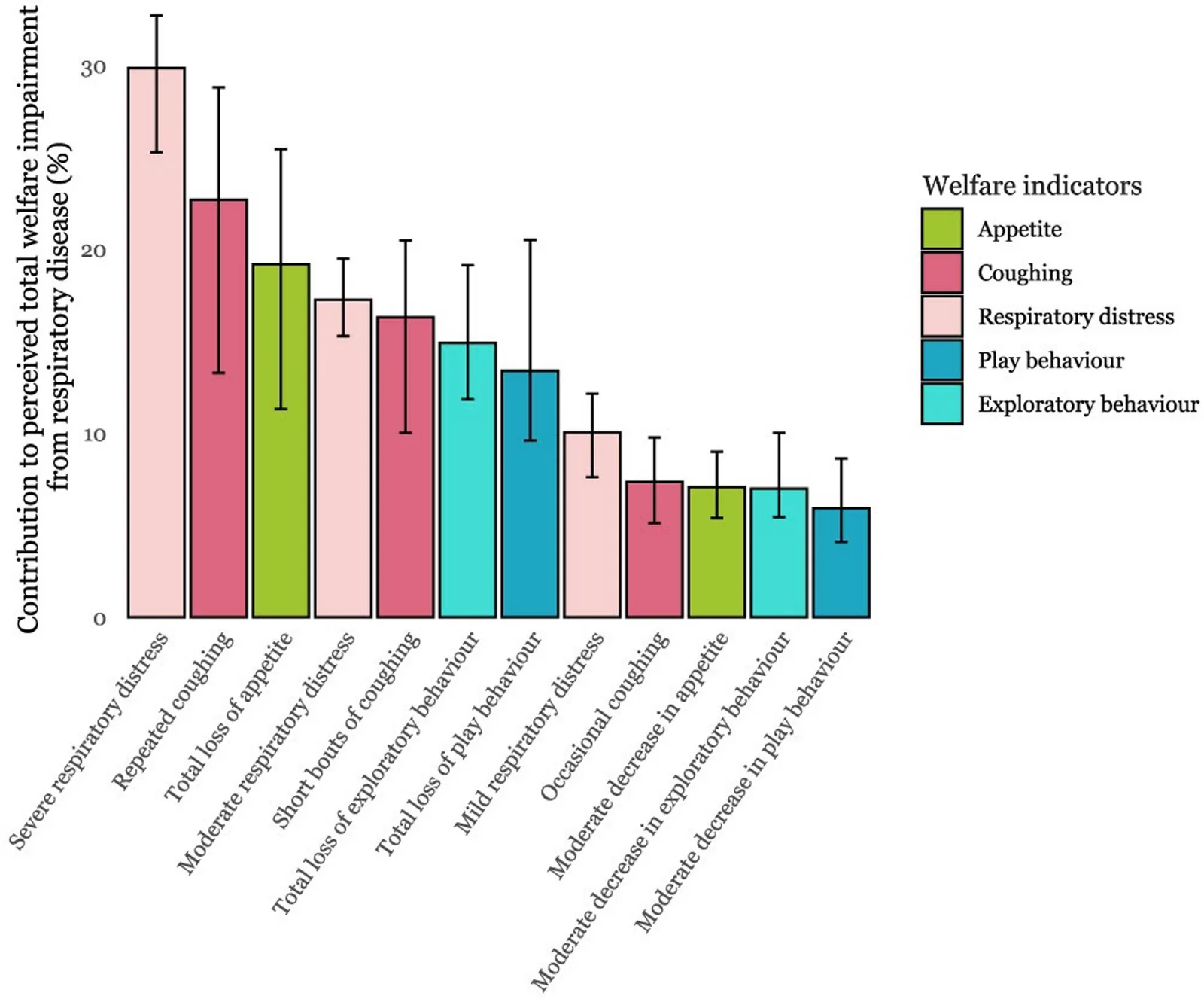
Relative contribution of welfare indicator levels to perceived pig welfare impairment from respiratory disease. Values are expressed relative to the optimal scenario (first level of each indicator). The bars represent median values, with error bars representing the 2.5–97.5th percentile range across respondents

Figure 2 presents the relative contributions of specific welfare indicator levels to perceived pig welfare impairment from respiratory disease, with all other indicators held at their optimal levels. The underlying zero-centred disutility values are provided in Supplementary File Table 4. Figure 2 indicates that severe respiratory distress was perceived as the largest contributor to pig welfare impairment from respiratory disease, with an estimated contribution of 29.8%. This was followed by repeated coughing (+ 22.7%), total loss of appetite (+ 19.2%), moderate respiratory distress (+ 17.2%), and short bouts of coughing (+ 16.3%). In contrast, moderate reductions in play and exploratory behaviour, moderate loss of appetite, and occasional coughing were perceived to contribute comparatively less to welfare impairment from respiratory disease, each associated with less than a 10% increase in welfare impairment from respiratory disease. The 2.5–97.5th percentile range overlapped for several indicators, indicating variability in respondents’ perceptions of their contribution to welfare impairment from respiratory disease. Statistically significant differences between researchers and veterinarians were limited to two indicator levels: occasional coughing and total loss of play behaviour. For occasional coughing, researchers assigned higher contributions to welfare impairment from respiratory disease than veterinarians (*P* < 0.05), whereas for total loss of play behaviour, veterinarians assigned higher contributions than researchers (*P* < 0.05).

**Table 3:** Mean relative importance scores of welfare indicators per respondent group

Domain	Welfare indicator	Mean relative importance score % (2.5–97.5th percentile range)	
		Animal welfare researchers	Practising swine veterinarians
Nutrition	Appetite	18.7 (11.8–25.4)	19.8 (12.6–25.0)
Health	Coughing	24.0 (16.9–29.4)	20.8 (13.5–28.0)
	Respiratory distress	30.3 (27.3–33.0)	29.3 (24.6–32.5)
Behaviour	Play behaviour	12.3 (9.6–16.2)	14.9 (11.5–22.9)
	Exploratory behaviour	14.7 (11.8–18.0)	15.3 (12.5–19.9)

Individual relative importance scores were calculated by taking the range of part-worth disutilities for each indicator, dividing it by the sum of ranges across all welfare indicators for that respondent, and expressing the result as a percentage [14]. Higher relative importance scores indicate greater relevance of the indicator to overall perceived welfare impairment from respiratory disease

### Illustrative exercise: perceived welfare impairment by single pathogen infections

**Fig. 3:**
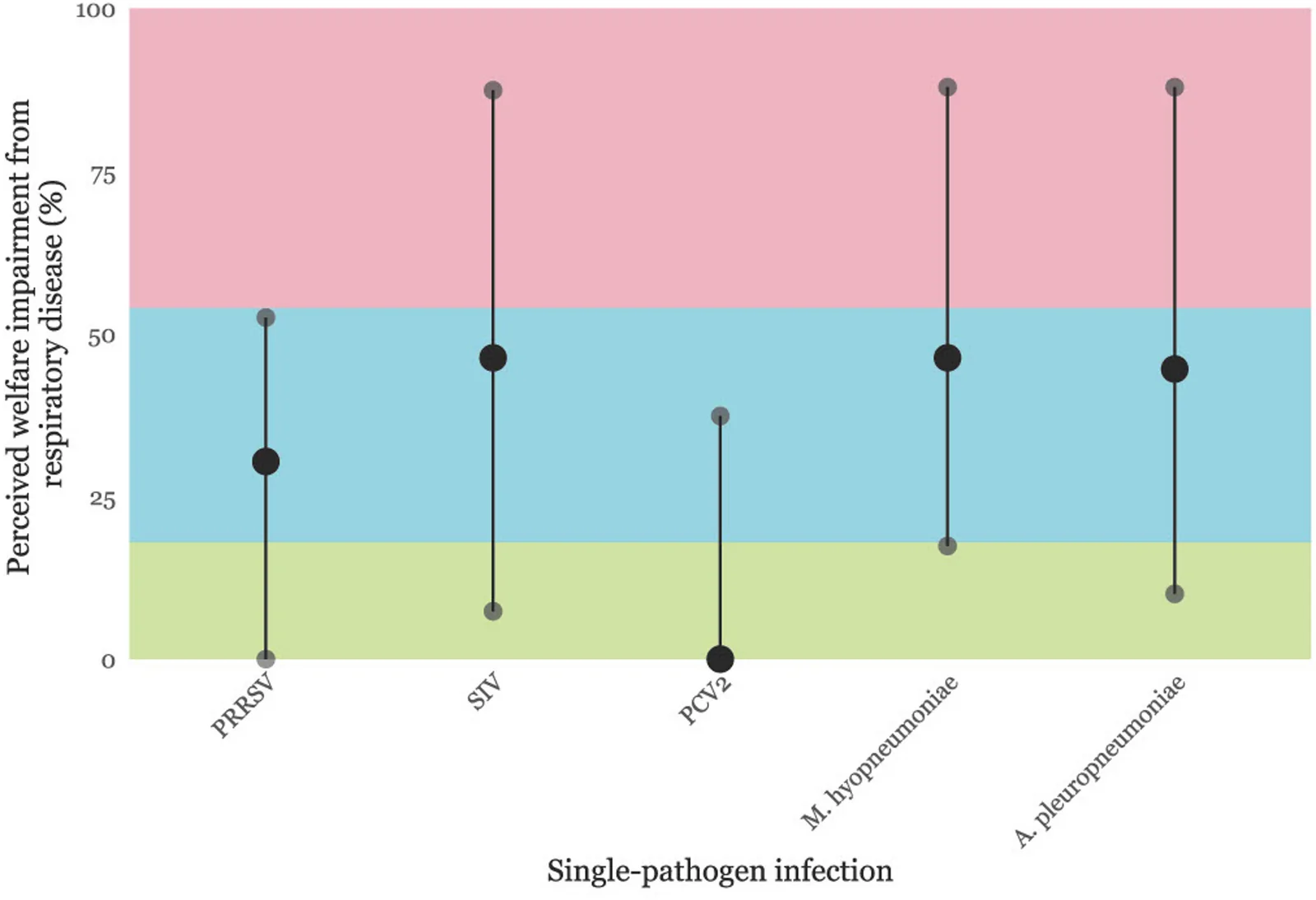
Perceived pig welfare impairment attributable to respiratory disease for single-pathogen infections. The profiles were derived from expert elicitation rather than survey data and are intended for illustrative purposes only. Estimates represent welfare impairment from respiratory disease for an individual pig at a single point in time and do not account for disease duration. Large dots indicate the most likely scenario, while smaller dots represent minimum and maximum scenarios. The scale ranges from 0% (optimal scenario; no welfare impairment from respiratory disease) to 100% (most severe welfare impairment from respiratory disease). Shaded areas denote mild (green; 0–18%), moderate (blue; 18–54%) and severe (pink; 54–100%) severity scenarios of respiratory disease. PRRSV = porcine reproductive and respiratory syndrome virus; SIV = swine influenza virus; PCV2 = porcine circovirus type 2; M. hyopneumoniae = *Mycoplasma hyopneumoniae*; A. pleuropneumoniae = *Actinobacillus pleuropneumoniae*

Figure 3 illustrates the perceived ranges of welfare impairment corresponding to different respiratory disease severity levels (shaded areas), along with the relative welfare impairment associated with minimum, most likely, and maximum manifestation profiles for five single-pathogen infections. These profiles are for illustrative purposes only and should not be interpreted as observed outcomes.

Based on these evaluations, mild, moderate, and severe respiratory disease corresponded to 0–18%, 18–54%, and 54–100% welfare impairment from respiratory disease, respectively. Respiratory disease caused by single infections with SIV, *M. hyopneumoniae*, and *A. pleuropneumoniae* was perceived to impair welfare similarly, with most likely impairment levels corresponding to moderate respiratory disease severity (≈ 50% on the study-specific impairment scale) but with the potential to reach severe levels (≈ 90%). In contrast, single infections with PRRSV and PCV2 were not perceived to cause severe welfare impairment from their respiratory effects, with PCV2 in particular associated with a minimal or mild impairment.

## Discussion

This study presents an application of ACA to quantify perceived welfare impairment associated with respiratory disease in fattening pigs. By including indicators from the nutrition, health, and behaviour domains, this work provides a novel quantitative application of the Five Domains Model in disease-related welfare assessment. Although ACA has been used in animal welfare research previously [12, 13], this study is the first to explicitly compare the perspectives of animal welfare researchers and practising swine veterinarians, and to illustrate how ACA-derived disutilities can be applied to quantify welfare impairment under different respiratory disease scenarios.

Across respondent groups, indicators from the health domain received the highest relative importance scores, indicating that their most severe levels were perceived as having the greatest detrimental impact on pig welfare. Severe respiratory distress was perceived as the largest contributor to pig welfare impairment from respiratory disease, followed by repeated coughing, total loss of appetite, moderate respiratory distress, and short bouts of coughing. Behavioural indicators were ranked lowest, although they were still considered relevant contributors. Animal welfare researchers and practising swine veterinarians showed broadly similar response patterns, with statistically significant differences limited to two indicator levels. Animal welfare researchers assigned higher welfare impairment to occasional coughing, whereas veterinarians assigned higher impairment to total loss of play behaviour (both *P* < 0.05), but these differences could not be fully explained. Exploratory comparisons within the animal welfare researcher group, contrasting those with higher versus lower levels of experience in animal welfare research, did not reveal any statistically significant differences in response patterns (results not shown). It is important to emphasise that our results reflect human perceptions of pig welfare impairment rather than direct animal-based measures. Nevertheless, the use of expert judgement is not uncommon in animal welfare science. Approaches such as Qualitative Behaviour Assessment (QBA) rely on human observers to interpret behavioural expressions of animals, and research has shown that such assessments can be both reliable and repeatable, even among observers with limited training [19, 20]. Although QBA involves direct observation rather than survey-based judgements, these findings suggest that human evaluations of animal welfare can provide consistent and meaningful insights. In this context, the strong agreement observed between respondent groups in the present study may indicate that both animal welfare researchers and practising swine veterinarians share broadly similar mental models, or internal frameworks, for understanding and predicting how respiratory disease affects pig welfare.

A few methodological limitations should be considered when interpreting and using the resulting relative importance scores and welfare impairment estimates. Within the conjoint theory, selected indicators are ideally independent, such that each reflects a distinct aspect of the evaluated profile. In the present context, however, clinical signs of respiratory disease and associated behavioural changes are biologically interrelated through the underlying pathogenesis. As a result, respondents’ evaluations of individual indicators may have been influenced by the other indicators presented within a profile. This challenges the ACA assumption of independence and may lead to partially biased part-worth estimates, where the relative importance assigned to one indicator depends on the presence or severity of others. Consequently, relative importance scores may be inflated for more prominent indicators, while the contribution of correlated indicators may be underestimated. Nevertheless, the relative ranking of the highest- and lowest-ranked indicators and levels is likely to be robust to these effects, even if the exact disutility estimates may be affected. A related consideration concerns the exclusion of implausible indicator combinations. In the present study, pig health experts were involved in selecting indicators that were as independent from each other as feasible and in restricting the paired-comparison tasks to biologically plausible combinations of indicator levels. Although standard ACA guidelines typically discourage restricting combinations [21], this decision was made to enhance interpretability and decrease response noise [15]. This restriction, however, reduces variation in the combinations presented and may therefore limit the ability to fully disentangle the individual contributions of the indicators, thereby influencing the estimated part-worth disutilities and derived relative importance scores. At the same time, the described interrelations reflect the biological reality of respiratory disease. When welfare impairment is evaluated for realistic profiles (i.e. where correlated indicator levels co-occur), such as illustrated in the present study for single-pathogen infections, the resulting welfare impairment estimates remain meaningful, as they align with the way respondents interpret such combinations in practice.

Moreover, respondents were required to evaluate welfare indicators based on written descriptions rather than direct observation. Although all participating veterinarians and most animal welfare researchers reported having relevant experience with pigs, variation in familiarity with pig behaviour could influence how consistently such indicators are interpreted. Future studies are recommended to strengthen elicitation exercises by providing standardised visual prompts (e.g., video examples of behaviour and clinical signs) to support consistent interpretation across respondents. In addition, as this study does not explicitly assess the mental state within the Five Domains Model, future work could use such visual prompts to facilitate integration of ACA with QBA, incorporating evaluation of behavioural expressions associated with specific welfare profiles and thus enabling comparison between perceived welfare impairment based on the physical domains and affective states inferred from these behavioural expressions.

Finally, we applied a 0–100% welfare impairment scale to improve interpretability, which represents a novel way of reporting ACA-derived outcomes compared to previous studies [12, 13]. However, it is important to emphasise that a 100% welfare impairment on our scale does not represent the maximum possible welfare compromise in an absolute sense, but rather the most severe profile defined within the respiratory indicators included in this study. As such, the scale should be interpreted as a bounded measure of welfare impairment within a defined respiratory-disease context, structured according to indicators aligned with the nutrition, health and behaviour domains of the Five Domains Model. This provides a quantitative implementation of the Five Domains approach for comparing respiratory disease-related welfare impacts, but does not extend to an assessment of total welfare compromise. In future studies, respondents could be explicitly asked to relate profiles defined within the study to what they consider the absolute maximum possible welfare impairment in pigs. Such an approach would facilitate interpretation of how the assessed profiles relate to broader multifactorial welfare compromise, including welfare issues such as heat stress, lameness, gastro-enteric disorders and skin or tail lesions [22].

The outcomes of this study have several potential applications. Firstly, our findings may further support and refine existing welfare assessment protocols, such as the Welfare Quality^®^ Protocol [23]. Within the “Good Health – Absence of disease” principle, respiratory indicators such as coughing and respiratory distress are currently included as measures of welfare using relatively coarse classifications. For example, coughing in sows or individual pigs is recorded as a binary measure indicating either the absence or presence of coughing within a five-minute observation period. While such measurements allow for practical on-farm assessment, they do not capture variation in welfare impairment relating to the severity of respiratory signs. Results of the present study suggest that there is a distinct difference in welfare impairment across levels of coughing and respiratory distress. Thus, distinguishing between different levels of these indicators, and weighting them accordingly, could improve the representation of welfare impairment due to respiratory health problems in existing protocols. Secondly, our results provide a foundation for integrating welfare metrics into quantitative modelling and disease burden evaluations. By constructing disease profiles and rescaling total disutility, welfare impairment in an individual pig at a given moment could be expressed on a scale from 0% (optimal scenario; no welfare impairment from respiratory disease) to 100% (most severe welfare impairment from respiratory disease). In individual-based epidemiological models that simulate prevalence, duration, and progression of respiratory disease [24], batch-level welfare impairment could be estimated by linking modelled disease states to combinations of welfare indicator levels. In this way, welfare profiles can be constructed for each phase of infection, allowing the development of individual or group-averaged welfare impairment to be tracked over the course of the simulation. This approach also allows evaluation of the welfare benefits of interventions such as vaccination, biosecurity improvements, or treatment protocols. However, the present study focused on pigs aged 8–10 weeks, due to available literature on play and exploratory behaviours in pigs affected by respiratory disease [6]. Recent evidence suggests that play behaviour declines over the fattening period, whereas other behaviours may increase [25], thereby altering the definition of “optimal” behavioural expression. Therefore, caution is warranted when applying the results on behavioural indicators to pigs across the fattening period. Finally, the study outcomes could be applied to identify and prioritise key welfare issues related to respiratory disease. We explored this through the illustrative exercise estimating perceived welfare impairment from respiratory disease for five single-pathogen infections. Despite its exploratory nature, this exercise illustrates how indicator disutilities could be combined to facilitate quantitative comparison of welfare impacts across disease scenarios. Importantly, some pathogens likely cause welfare impacts outside the included indicators, meaning that the current profiles may underestimate total impairment. For example, systemic pathogens such as PRRSV and PCV2 were evaluated only through respiratory indicators, thereby excluding potential non-respiratory health impacts. Moreover, highly severe disease scenarios may lead to suppression of observable clinical signs, as seen in peracute disease caused by *A. pleuropneumoniae* where pigs may be found dead without showing prior respiratory signs [26]. In addition, expert judgements may have been influenced by difficulties in conceptualising single-pathogen scenarios, given that respiratory disease in practice is often characterised by mixed infections, as well as by perceptions of disease duration, virulence, and routine intervention practices, which vary across pathogens, production systems and countries. In combination with the limited number of participating experts, this exercise should be interpreted as a methodological demonstration of how disutilities can be combined into composite welfare profiles, rather than as a definitive ranking of pathogen-specific welfare impacts.

## Conclusions

This study demonstrates the application of survey-based adaptive conjoint analysis to quantify perceptions of welfare impairment from respiratory disease in fattening pigs. Health indicators were consistently perceived as strong contributors to welfare impairment, while behavioural and nutrition-related indicators also remained relevant. Similar patterns were observed across respondent groups, indicating broadly consistent perceptions of the relative importance of welfare indicators between practising swine veterinarians and animal welfare researchers. An illustrative exercise demonstrated how ACA-derived disutilities may be combined to estimate and compare welfare impairment across different disease scenarios. Future elicitation exercises would benefit from explicitly incorporating typical mixed-infection patterns, clearly specifying disease stages (e.g. peak severity), environmental conditions and intervention practices, and including a broader range of welfare indicators. In addition, the findings of this study may inform refinement of welfare assessment protocols and support integration of welfare metrics into epidemiological models. However, the results should be interpreted as perceived welfare impairment within the selected respiratory disease indicator set rather than as direc measures of overall pig welfare. Future work should integrate observational approaches, include an assessment of the mental state, and extend the framework to broader, multifactorial welfare contexts.

## Supplementary Information

Below is the link to the electronic supplementary material.

Supplementary File 1

## Data Availability

The datasets used and/or analysed during the current study are available from the corresponding author on reasonable request.

## Declarations

### Declaration of AI

During the preparation of this work, the authors used ChatGPT (GPT-5) to assist with data analysis and improve readability and language. After using this tool, the authors reviewed and edited the content as needed and take full responsibility for the content of the publication.

## Abbreviations

ACA: Adaptive conjoint analysis
*A.**pleuropneumoniae*: *Actinobacillus pleuropneumoniae*
*M. hyopneumoniae*: *Mycoplasma hyopneumoniae*
PCV2: porcine circovirus type 2
PRDC: porcine respiratory disease complex
PRRSV: porcine reproductive and respiratory syndrome virus
QBA: Qualitative Behaviour Assessment
SIV: swine influenza virus

## References

[1] Pieters M, Canturri A. Respiratory Diseases in Pigs. In: Gross JJ, editor. Production Diseases in Farm Animals. Cham: Springer International Publishing; 2024. pp. 305–16.

[2] Saade G, Deblanc C, Bougon J, Marois-Créhan C, Fablet C, Auray G, et al. Coinfections and their molecular consequences in the porcine respiratory tract. Vet Res. 2020;51(1):80. 10.1186/s13567-020-00807-8 PMC7296899 32546263

[3] Mellor DJ. Operational Details of the Five Domains Model and Its Key Applications to the Assessment and Management of Animal Welfare. Anim (Basel). 2017;7(8):60. 10.3390/ani7080060. PMC5575572 28792485

[4] Petri FAM, Ferreira GC, Arruda LP, Malcher CS, Storino GY, Almeida HMS, et al. Associations between Pleurisy and the Main Bacterial Pathogens of the Porcine Respiratory Diseases Complex (PRDC). Anim (Basel). 2023;13(9):1493. 10.3390/ani13091493. PMC10177087 37174529

[5] Pastorelli H, van Milgen J, Lovatto P, Montagne L. Meta-analysis of feed intake and growth responses of growing pigs after a sanitary challenge. Animal. 2012;6(6):952–61. 10.1017/S175173111100228X. 22558966

[6] Steinerová K, Harding JCS, Parker SE, Wilson HL, Nery Finatto A, Seddon YM. Rearing pigs with play opportunities: the effects on disease resilience in pigs experimentally inoculated with PRRSV. Front Veterinary Sci. 2024;11–2024. 10.3389/fvets.2024.1460993. PMC11443507 39355142

[7] Algers B, Blokhuis HJ, Broom DM, Costa P, Domingo M, Greiner M, et al. Opinion of the Scientific Panel on Animal Health and Welfare on a request from the Commission on animal health and welfare in fattening pigs in relation to housing and husbandry. EFSA; 2007.

[8] Válková L, Voslářová E, Conte F, Večerek V. Animal welfare in terms of lung health in cattle, pigs, sheep and goats assessed at slaughter. Acta Vet Brno. 2024;93(1):77–85.

[9] Pessoa J, da Costa MR, Manzanilla EG, Norton T, McAloon C, Boyle L. Managing respiratory disease in finisher pigs: Combining quantitative assessments of clinical signs and the prevalence of lung lesions at slaughter. Prev Vet Med. 2021;186:105208. 10.1016/j.prevetmed.2020.105208 33310195

[10] Nielsen SS, Houe H, Denwood M, Nielsen LR, Forkman B, Otten ND, et al. Application of Methods to Assess Animal Welfare and Suffering Caused by Infectious Diseases in Cattle and Swine Populations. Anim (Basel). 2021;11(11):3017. 10.3390/ani11113017. PMC8614463 34827750

[11] Rioja-Lang F, Bacon H, Connor M, Dwyer CM. Prioritisation of animal welfare issues in the UK using expert consensus. Vet Rec. 2020;187(12):490. 10.1136/vr.105964. PMC7848064 32631849

[12] Zaghen F, van der Voort M, Rodenburg TB, Hogeveen H, Jorritsma R, Steeneveld W. Quantifying welfare impact of bovine respiratory disease in dairy calves via expert elicitation. Italian J Anim Sci. 2025;24(1):2541–51. 10.1080/1828051x.2025.2585603.

[13] Edwardes F, van der Voort M, Rodenburg TB, Hogeveen H. A new approach and insights on modelling the impact of production diseases on dairy cow welfare. Animal. 2024;18(4):101056. 10.1016/j.animal.2023.101056. 38460468

[14] Orme BK. Getting Started with Conjoint Analysis: Strategies for Product Design and Pricing Research. LLC, Madison: Research; 2014.

[15] Eggers F, Sattler H, Teichert T, Völckner F. Choice-based conjoint analysis. Handbook of market research. Springer; 2021. pp. 781–819.

[16] Sawtooth software I. Lighthouse studio. 9.16.16 ed2026.

[17] Sawtooth Software I. The ACA/Web v6.0 technical paper. 2007. https://sawtoothsoftware.com/resources/technical-papers/aca-technical-paper. Accessed 10-07-2026.

[18] Team RC. A Language and Environment for Statistical Computing. Version 4.5.0 ed. Vienna, Austria: R Foundation for Statistical Computing; 2025.

[19] Wickham S, Collins T, Barnes A, Miller D, Beatty D, Stockman C, et al. Qualitative behavioral assessment of transport-naïve and transport-habituated sheep. J Anim Sci. 2012;90(12):4523–35. 10.2527/jas.2010-3451 22829616

[20] Stockman C, Collins T, Barnes A, Miller D, Wickham S, Beatty D, et al. Qualitative behavioural assessment and quantitative physiological measurement of cattle naïve and habituated to road transport. Anim Prod Sci. 2011;51(3):240–9.

[21] Sawtooth software lighthouse studio. Specifying prohibitions in ACA. 2026. https://sawtoothsoftware.com/help/lighthouse-studio/manual/cbc-prohibitions.html. Accessed 06-03-2026.

[22] Nielsen SS, Alvarez J, Bicout DJ, Calistri P, Canali E, Drewe JA, et al. Welfare of pigs on farm. Efsa j. 2022;20(8):e07421. 10.2903/j.efsa.2022.7421. PMC9405538 36034323

[23] Welfare Quality^®^. Welfare Quality^®^ assessment protocol for pigs (sows and piglets, growing and finishing pigs). Lelystad, Netherlands: Welfare Quality^®^ Consortium; 2009.

[24] Boeters M, Garcia-Morante B, Picault S, van Schaik G, Sibila M, Segalés J, et al. An integrated individual-based model of transmission, clinical outcomes, and economic impact of Mycoplasma hyopneumoniae infection in a commercial pig fattening unit. animal. 2026;20(4):101786. 10.1016/j.animal.2026.101786. 41855918

[25] Puttkammer N, Czycholl I. Play in fattening pigs: prevalence and potential as indicator of positive welfare. J Appl Anim Welfare Sci. 2025:1–20. 10.1080/10888705.2025.2596199. 41362234

[26] Stringer OW, Li Y, Bossé JT, Langford PR. JMM Profile: Actinobacillus pleuropneumoniae: a major cause of lung disease in pigs but difficult to control and eradicate. J Med Microbiol. 2022;71(3). 10.1099/jmm.0.001483. PMC9176268 35262474

[27] Trindade PHE, de Araújo AL, Luna SPL. Weighted pain-related behaviors in pigs undergoing castration based on multilevel logistic regression algorithm. Appl Anim Behav Sci. 2023;265:106002. 10.1016/j.applanim.2023.106002.

[28] Roberts E, Hammer JM, Lechtenberg K. Investigation of tiamulin hydrogen fumerate in-feed antibiotic for the control of porcine respiratory disease complex that includes Mycoplasma hyopneumoniae. J Swine Health Prod. 2011;19(4):218–25.

[29] Steinerová K, Parker SE, Brown JA, Seddon YM. The promotion of play behaviour in grow-finish pigs: The relationship between behaviours indicating positive experience and physiological measures. Appl Anim Behav Sci. 2024;275:106263. 10.1016/j.applanim.2024.106263.

