# Supplementary material for: Quantifying welfare impairment from respiratory disease in fattening pigs using adaptive conjoint analysis
Source: Porcine Health Manag. 2026 Aug 14;12:54. doi: 10.1186/s40813-026-00533-1 (PMC13753133; doi:10.1186/s40813-026-00533-1)
Supplement: Supplementary file 1 [file 40813_2026_533_MOESM1_ESM.docx]

SUPPLEMENTARY FILE

**Quantifying welfare impairment from respiratory disease in fattening pigs using adaptive conjoint analysis**

Marloes Boeters, Beatriz Garcia-Morante, Tijs Tobias, T. Bas Rodenburg, Carla Correia-Gomes, Maria Costa, Joaquim Segales, Jonathan Rushton, Gerdien van Schaik, Wilma Steeneveld

*Journal: Porcine Health Management*

**Supplementary File Table 1. Combinations of welfare indicator levels excluded from paired-comparison trade-off questions (stage 2).**

|  | Appetite | Coughing | Respiratory distress | Play behaviour | Exploratory behaviour |
| --- | --- | --- | --- | --- | --- |
| Appetite |  | None | 1 + 3; 1 + 4 | 2 + 1; 3 + 1;  3 + 2 | 1 + 3; 3 + 1 |
| Coughing |  |  | None | 4 + 1 | 4 + 1 |
| Respiratory distress |  |  |  | 3 + 1; 4 + 1;  4 + 2 | 3 + 1; 4 + 1;  4 + 2 |
| Play behaviour |  |  |  |  | 3 + 1; 1 + 3 |
| Exploratory behaviour |  |  |  |  |  |

Excluded combinations were identified by three pig health experts as indicator level combinations considered highly unlikely to occur in practice. Numbers refer to the coded levels of each welfare indicator (see Table 1 or Table S4 for definitions). The first number corresponds to the row indicator level and the second number to the column indicator level (e.g., “1+3” indicates level 1 of the row indicator combined with level 3 of the column indicator).

**Supplementary File Table 2. Selection of levels per severity scenario of respiratory disease.**

| Severity scenario | Selected level per welfare indicator | | | | |
| --- | --- | --- | --- | --- | --- |
|  | Appetite | Coughing | Respiratory distress | Play behaviour | Exploratory behaviour |
| Optimal | 1 | 1 | 1 | 1 | 1 |
| Mild | 1 | 2 | 2 | 1 | 1 |
| Moderate | 2 | 3 | 3 | 2 | 2 |
| Severe | 3 | 4 | 4 | 3 | 3 |

Numbers refer to the coded levels of each welfare indicator (see Table 1 or Table S4 for definitions).

**Supplementary File Table 3. Selection of levels per single pathogen infection scenario.**

| Pathogen | Most likely level (plausible range) | | | | |
| --- | --- | --- | --- | --- | --- |
|  | Appetite | Coughing | Respiratory distress | Play behaviour | Exploratory behaviour |
| PRRSV | 2 (1-2) | 2 (1-2) | 2 (1-3) | 2 (1-2) | 1 (1-3) |
| SIV | 2 (1-3) | 3 (2-4) | 2 (1-3) | 2 (1-3) | 2 (1-3) |
| PCV2 | 1 (1-2) | 1 (1-2) | 1 (1-2) | 1 (1-2) | 1 (1-2) |
| MHYO | 2 (1-2) | 3 (2-4) | 2 (2-4) | 2 (1-3) | 2 (1-3) |
| APP | 2 (1-2) | 2 (1-4) | 3 (2-4) | 2 (1-3) | 2 (1-3) |

Profiles per single pathogen infection scenario were constructed based on elicitation from five pig health experts, who independently defined the most likely welfare indicator levels and plausible (95%) ranges of disease manifestations in an individual pig at a single point in time. Numbers refer to the coded levels of each welfare indicator (see Table 1 or Table S4 for definitions). PRRSV = porcine reproductive and respiratory syndrome virus; SIV = swine influenza virus; PCV2 = porcine circovirus type 2; MHYO = *Mycoplasma hyopneumoniae*; APP = *Actinobacillus pleuropneumoniae*.

**Supplementary File Table 4. Zero-centred part-worth disutilities per welfare indicator level and respondent group**

| Domain | Welfare indicator and level | Average disutilities (SD) | |
| --- | --- | --- | --- |
|  |  | *Animal welfare researchers* | *Veterinarians* |
| Nutrition | Appetite  1. Normal (optimal)  2. Moderate decrease  3. Total loss | -42.9 (7.7)  -7.9 (6.1)  50.7 (12.8) | -45.0 (8.7)  -9.2 (6.2)  54.2 (13.9) |
| Health | Coughing  1. Absence (optimal)  2. Occasional  3. Short bouts  4. Repeated coughing | -60.7 (8.6)  -23.4 (6.8)  24.7 (5.3)  59.4 (10.0) | -53.9 (13.2)  -17.9 (5.9)  22.0 (5.3)  49.8 (13.8) |
|  | Respiratory distress  1. Absence (optimal)  2. Mild  3. Moderate  4. Severe | -72.2 (3.6)  -21.5 (3.8)  14.4 (3.7)  79.3 (6.2) | -70.3 (4.5)  -20.8 (4.4)  15.5 (3.5)  75.7 (8.2) |
| Behaviour | Play behaviour  1. Normal (optimal)  2. Moderate decrease  3. Total loss | -29.7 (4.8)  -2.0 (2.4)  31.7 (6.1) | -35.8 (7.7)  -3.4 (3.4)  39.2 (10.4) |
|  | Exploratory behaviour  1. Normal (optimal)  2. Moderate decrease  3. Total loss | -35.9 (3.8)  -1.8 (3.5)  37.6 (5.6) | -37.4 (6.1)  -1.2 (3.2)  38.6 (6.6) |
